# Supplementary material for: The role of geography in the complex diffusion of innovations
Source: Sci Rep. 2020 Sep 15;10:15065. doi: 10.1038/s41598-020-72137-w (PMC7492253; doi:10.1038/s41598-020-72137-w)
Supplement: Supplementary file 1 — Supplementary Information. [file 41598_2020_72137_MOESM1_ESM.pdf]

# Supplementary Information to
# The role of geography in the complex diffusion of innovations

Balázs Lengyel[a,b,c,d,*], Eszter Bokányi[c,d], Riccardo Di Clemente[a,e,f], János Kertész[g], and Marta C. González[a,h,i]

[a]Massachusetts Institute of Technology, Department of Civil and Environmental Engineering, Cambridge MA, 02139, USA
[b]International Business School Budapest, Budapest, 1037, Hungary
[c]Agglomeration and Social Networks Lendület Research Group, Centre for Economic- and Regional Studies, Institute of Economics, Budapest, 1097, Hungary
[d]Corvinus University of Budapest, Institute of Advanced Studies, Budapest, 1093, Hungary
[e]University of Exeter, Computer Science Department, Exeter, EX4 4QF, United Kingdom
[f]University College London, The Bartlett Centre for Advanced Spatial Analysis, London, WC1E 6BT, United Kingdom
[g]Central European University, Department of Network and Data Science, Budapest, 1051, Hungary
[h]University of California at Berkeley, Department of City and Regional Planning, Berkeley CA, 94720, USA
[i]Energy Analysis and Environmental Impacts Division, Lawrence Berkeley National Laboratory, Berkeley Ca, 94720, USA
[*]Corresponding address: lengyel.balazs@krtk.mta.hu

## Supporting Information 1: Spatial diffusion and churn over the product life-cycle

Video on spatial diffusion and churn of iWiW. Nodes denote towns and links represent invitations sent across towns between 2002 and 2012 on a monthly basis. The size of nodes illustrates the number of users who registered in the town by the given month and the color depicts the share of those registered users who still logged in. Adoption started in Budapest (the capital) and was followed first in its surroundings and other major regional subcenters. The vast majority of invitations have been sent from Budapest in the initial phase of diffusion and subcenters started to transmit spreading when diffusion speeded up in the middle of the life-cycle. A decisive fraction of users logged in to the website even after Facebook entered the country in 2008. Collective churn started in 2010 and the rate of active users dropped quickly in most of the towns. Exceptions are small villages in the countryside, where people have difficulties to adopt new waves of social media innovation.

For the video on spatial diffusion and churn, go to `https://vimeo.com/251494015`

## Supporting Information 2: Correlation of Bass model predictions and geographical characteristics

Prediction Error correlates negatively with peak of adoption indicating that Bass prediction of peaks works better in towns that adopt late. Town size and Distance from the Capital are negatively correlated with each other ($\rho = -0.32$). We find that $q_i$ is significantly smaller in large towns than in small towns. There is a significant negative correlation between the month of Predicted Peak and $q_i$; while this correlation with $p_i$ is positive. Standard errors of $p_i$ and $q_i$ correlate strongly with the respective parameters. Further correlations of $SEq_i$ indicate that estimation of $q_i$ is significantly more accurate in towns where adoption peaks late but is less accurate in towns that are far from Budapest.

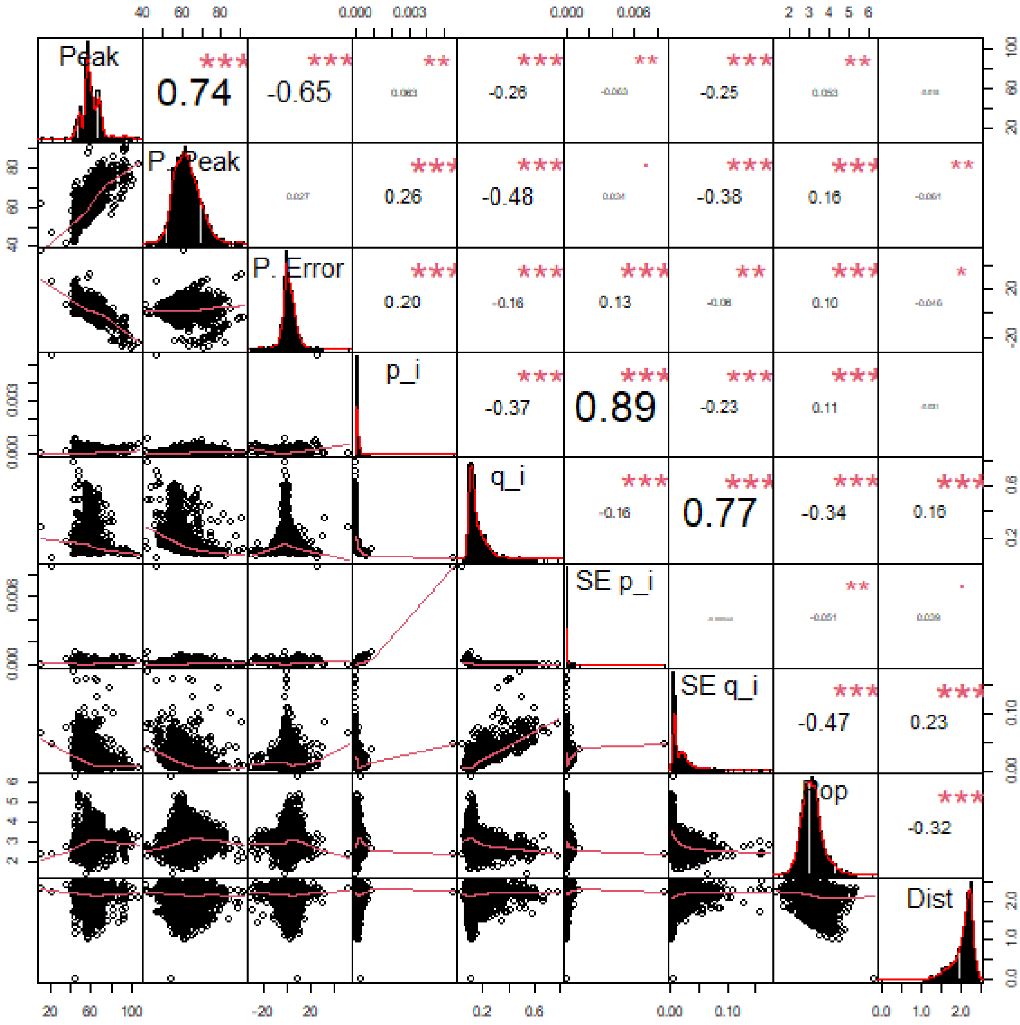


Figure S1: Pearson correlation coefficients of Bass model and geographical characteristics of towns. Peak denotes month of observed adoption peak in towns; P. Peak is the predicted month of adoption peak by the Bass model; P. Error is predicted month of adoption peak minus the empirical peak; $p_i$ and $q_i$ denote Bass model parameters; $SEp_i$ and $SEq_i$ are standard errors of the estimated parameters; Pop denotes $log_{10}$ of town population and Dist denotes $log_{10}$ kilometers from Budapest.

# Supporting Information 3: Network sampling for ABM

To model diffusion in the empirical social network, we sample the full network of 3 Million nodes by keeping the distribution of nodes according to locations and network communities. This is done by identifying the community structure of the full network with the Louvain algorithm and assigning every node into one community. Then, we take 5%, 10%, 20% samples and stop sampling when the p-value of the Kolmogorov-Smirnov test comparing both town and community distributions of the sampled and full node lists is larger than 0.95. Finally, we connect the nodes with ties that link them in the full network and exclude those nodes that are not part of the giant component.

In Table S1, we compare structural characteristics of the 5%, 10%, and 20% sample networks with the full network. Density of links in the sampled networks are on the same magnitude as the full network. However, the smaller sample we take the higher density. Global clustering (the ratio of closed triangles among all possible triangles) is identical across samples, which is around half of the full network. The fraction of links that connect individuals across towns are identical in the samples and the full network.

Table S1: Characteristics of sampled networks

| **Sample** | **5%** | **10%** | **20%** | **100%** |
|---|---|---|---|---|
| Nodes in Giant Component | 128,590 | 271,941 | 564,134 | 3,050,988 |
| Links | 675,227 | 2,712,588 | 10,799,507 | 279,708,125 |
| Density | $8.167 \times 10^{-5}$ | $7.33 \times 10^{-5}$ | $6.78 \times 10^{-5}$ | $6.01 \times 10^{-5}$ |
| Global clustering | 0.09 | 0.09 | 0.09 | 0.17 |
| Links across towns, % | 50.1% | 51.2% | 51.1% | 51.1% |

In Figure S2, we plot degree distribution and distance decay of connections for each sample and the full network. The sample degree distributions lack the high probability of low-degrees ($k < 10$) that is an interesting characteristic of the full network. Further, the probability of ties at short distances ($d < 10^{1.5}$) deviate positively from the generally observed distance decay in the full network. This deviation is present in the sample networks as well, but only to a lesser extent.

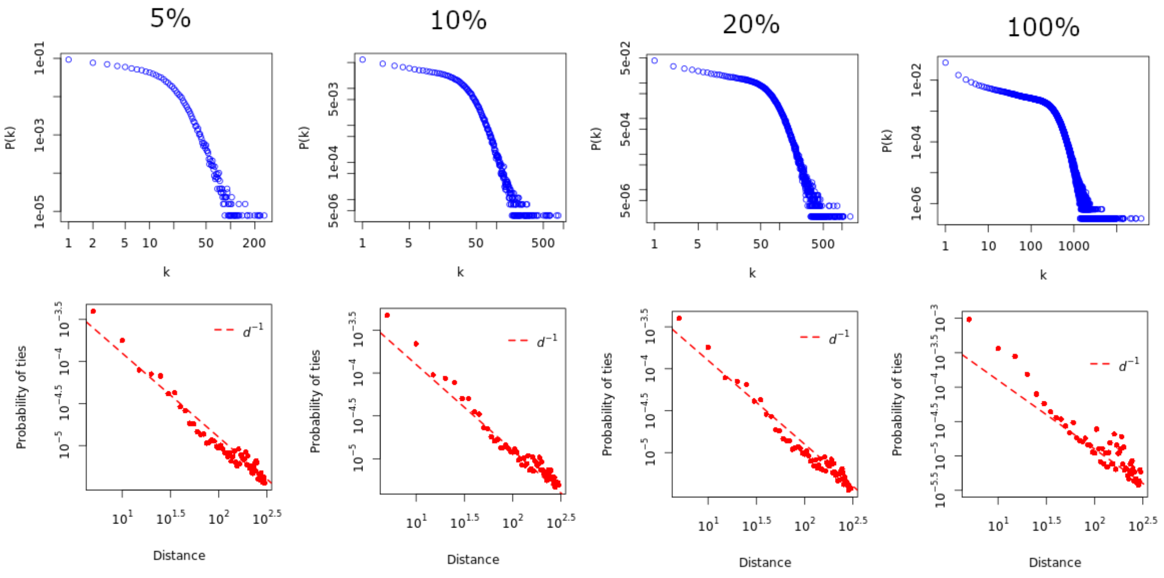


Figure S2: Degree distribution and distance decay in the sampled networks

In sum, by taking the 10% sample of the full network, we cannot fully represent the fraction of low degree nodes and short-distance linkages. Consequently, Density is higher and Global Clustering is lower in the sampled network than in the full network. In our understanding, this slight bias does not disturb the consistency of our findings, since urban scaling of adoption and distance decay of spreading have similar patterns in the full network and in the 10% sample we apply in the ABM.

# Supporting Information 4: Calibration of ABM parameters and their influence on adoption

## Fitting the ABM to the diffusion data

We fit our basic ABM model to the diffusion data using the method of Xiao et al. [1]. The first step in the fitting is finding the linear transformation between the macroscopic $p$ and $q$ parameters of the solution of the Bass differential equation (see Eq.1), and the microscopic $p^{ABM}$ and $q^{ABM}$ parameters that drive the neighborhood adoption in the ABM (see Eq.3).

$$\begin{pmatrix} p^{ABM} \\ q^{ABM} \end{pmatrix} = \underline{\underline{C}} \begin{pmatrix} p \\ q \end{pmatrix} + \underline{\varepsilon} \tag{S1}$$

First, to achieve this, we run several ABM models with all possible $(p, q)$ pairs, where $p \in \{0.25 \times 10^{-4}, 0.5 \times 10^{-4}, 0.75 \times 10^{-4}, \dots, 2 \times 10^{-4}\}$ and $q \in \{0.08, 0.1, 0.12, \dots, 0.2\}$, and fit the solution of the Bass equation with nonlinear least squares method to all of the adoption curves. Thus, we get the $(p, q)$ pairs corresponding to the $(p^{ABM}, q^{ABM})$ values, and by using OLS, we can fit both $\underline{\underline{C}}$ and $\underline{\varepsilon}$.

Second, we fit the Bass DE solution to the empirical adoption curve using again the nonlinear least squares method. From this fit, we get $\hat{p} = 0.0001570$ and $\hat{q} = 0.1047$. Substituting these values into Eq. S1, we get our initial estimates $p_0^{ABM} = 0.0001939$ and $q_0^{ABM} = 0.1191$ for the microscopic parameter values.

Starting out from this $(p_0, q_0)$ pair, we set up a grid in the $(p, q)$ parameter space with $\Delta p = 0.00001$ and $\Delta q = 0.01$. We are going to run ABMs corresponding to the $(p, q)$ pairs on this grid, and we characterize the goodness of fit of these ABMs with respect to the empirical data by calculating the sum of the squared deviation of the ABM adoption curve from the empirical adoption curve (SSE). We keep track of the already visited grid points, the SSE at each gridpoint, and the two gridpoints with the least SSEs so far. In each search step, we take these two points, and we run ABMs and calculate the corresponding SSEs for all of their neighboring gridpoints $(p \pm \Delta p, q \pm \Delta q)$ that we have not visited yet. Then, we determine the two new least SSE gridpoints, and continue the search. When there are no new neighbors for the two selected least SSE points that have not been visited yet, we stop the search, and select the parameter pair with the least SSE to be the parameters for the fitted ABM. Our final parameters after this optimization step are: $p_{opt}^{ABM} = 0.0001940, q_{opt}^{ABM} = 0.1191$.

## Selecting parameters to control for adoption threshold distribution

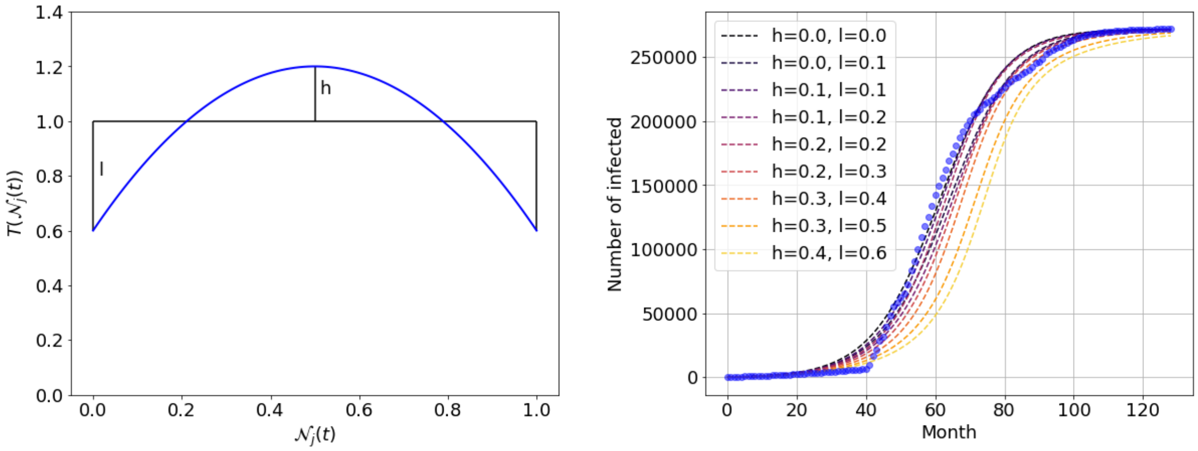


Figure S3: The transformation function for the modified ABM and parameter selection.

To find the optimal values of $h$ and $l$, we run ABMs with the previously calculated $p_{opt}^{ABM}$ and $q_{opt}^{ABM}$ parameters for different $(h, l)$ parameter pairs where $h \in \{0, 0.1, 0.2, \dots 1\}$ and $l \in \{0, 0.1, 0.2, \dots 1\}$. We then select the combinations for which the error was below the threshold $\log_{10} SSE < 10.2$. For these, we calculate the Pearson correlation of the peak adoption time of the largest towns (where population is greater than 5000) in the dataset. Then, as an alternative ABM model, we select $h = 0.2$ and $l = 0.2$,

since this combination gives the highest correlation $\rho = 0.12$ apart from the original $h = 0$, $l = 0$ model, for which $\rho = 0.14$. Figure S3 illustrates $T(x, h, l)$ from Eq. 5 (left) and CDF of ABM adoption considering various levels of $h$ and $l$ (right).

## The influence of transformation function on adoption probability

Figure S4 illustrates the transformation function $T(\mathcal{N}_j(t), h = 0.2, l = 0.2)$ and the empirical distribution of $\mathcal{N}_j(t)$ on the full network. In this paper, we do not aim to develop a perfect $T$ to weight adoption probability that can reproduce the empirical $\mathcal{N}_j(t)$ distribution. Instead, we intend to modify adoption probability in a simple way and motivated by the threshold distribution. Our approach captures the notion that $\mathcal{N}_j(t)$ peaks between 0.4 and 0.6 (Figure S4). However, empirical $\mathcal{N}_j(t)$ are relatively rare below 0.3 (these are high degree individuals, as reported in Figure 3B) that is not reflected by our $T$.

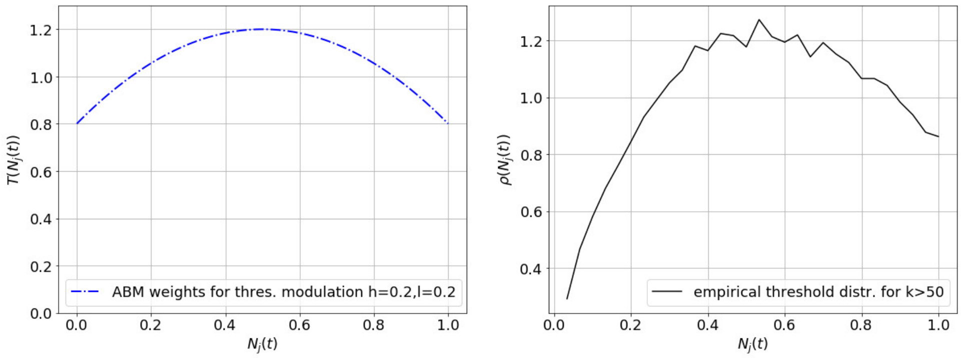


Figure S4: Threshold distribution and its modulation.

Substituting $T$ in Eq. 3 with Eq. 5 gives us adoption probability at $\mathcal{N}_j(t))$, which equals $\hat{p}^{\text{ABM}} + \mathcal{N}_j(t) \times \hat{q}^{\text{ABM}}$ in case $h = 0.0$ and $l = 0.0$ and $\hat{p}^{\text{ABM}} + (-1.6\mathcal{N}_j(t)^3 + 1.6\mathcal{N}_j(t)^2 + 0.8\mathcal{N}_j(t)) \times \hat{q}^{\text{ABM}}$ in case $h = 0.2$ and $l = 0.2$. We substitute $\hat{p}^{\text{ABM}}$ and $\hat{q}^{\text{ABM}}$ values and plot adoption probabilities as a function of $\mathcal{N}_j(t))$ in Figure S5 (left) and also their differences (right).

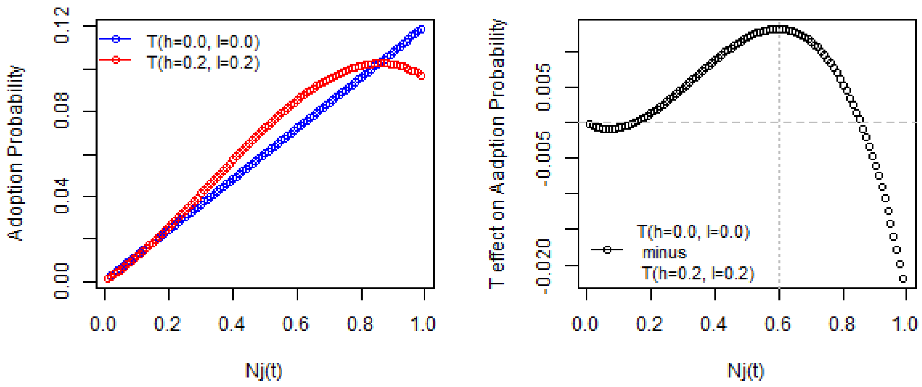


Figure S5: Linear and non-linear impact of neighbors on adoption probability.

Setting $h = 0.2$ and $l = 0.2$, instead of $h = 0.0$ and $l = 0.0$, slightly decreases adoption probability until $\mathcal{N}_j(t)) = 0.2$ but provides higher probability for $\mathcal{N}_j(t))$ values between 0.2 and 0.8. The additional probability of $h = 0.2$ and $l = 0.2$ is highest at $\mathcal{N}_j(t)) = 0.6$. Adoption probability of the $h = 0.2$ and $l = 0.2$ setting declines at $\mathcal{N}_j(t)) > 0.8$ such that probability at $\mathcal{N}_j(t)) = 1$ is approximately equal to the probability at $\mathcal{N}_j(t)) = 0.7$.

## Supporting Information 5: Urban scaling estimates with control variables

To understand, whether urban scaling of adoption is governed by demographic characteristics of towns, we run multiple OLS regressions with number of adopters across life-cycle stages as dependent variable. Independent variables include town population (log) and further measures that have been used in previous studies to predict adoption rate, or to investigate inequalities: development level (average income[2]), inequalities (Gini of income[3]), internet infrastructure and media presence (Telecom Composite Index, Number of TV, Number of School PC[2]), physical barriers of social interaction in towns (Rail-River Division [3]), segregation (Ethnic Entropy[3]), town hierarchy (Subregion Centre[2]).

We find a robust urban scaling coefficient reported in Figures 4A and 4C. Economic development of towns measured in average salary increases adoption at all phases of the life-cycle prediction; whereas development in terms of telecommunication infrastructure facilitates adoption in the Innovation phase only.

Table S2: Regression Results

| | *Dependent variable:* | | |
|---|---|---|---|
| | Innovator Users (log) | | |
| | (1) | (2) | (3) |
| Population (log) | 1.342*** | 1.297*** | 1.083*** |
| | (1.139, 1.546) | (1.183, 1.410) | (1.047, 1.118) |
| Average salary | 0.001*** | 0.0003** | 0.0001 |
| | (0.0003, 0.001) | (0.0001, 0.001) | (−0.00002, 0.0001) |
| Gini | 0.146 | 0.236 | 0.044 |
| | (−0.808, 1.100) | (−0.294, 0.766) | (−0.120, 0.208) |
| Telcom index | 0.082* | 0.041 | −0.016** |
| | (−0.012, 0.175) | (−0.011, 0.093) | (−0.032, −0.0004) |
| TV use | −0.002 | 0.003 | 0.001 |
| | (−0.009, 0.005) | (−0.001, 0.007) | (−0.0003, 0.002) |
| PC in school | −0.002 | −0.002 | 0.001 |
| | (−0.007, 0.004) | (−0.005, 0.001) | (−0.0004, 0.001) |
| RRDI | 0.067 | −0.014 | −0.021 |
| | (−0.167, 0.302) | (−0.147, 0.118) | (−0.062, 0.020) |
| Ethnic entropy | −0.454 | −0.249 | −0.006 |
| | (−1.422, 0.514) | (−0.795, 0.298) | (−0.175, 0.163) |
| Town | −0.070 | −0.022 | −0.012 |
| | (−0.250, 0.109) | (−0.120, 0.077) | (−0.042, 0.019) |
| Constant | −5.161*** | −4.259*** | −2.205*** |
| | (−6.740, −3.582) | (−5.145, −3.373) | (−2.480, −1.931) |
| Observations | 143 | 149 | 149 |
| $R^2$ | 0.726 | 0.869 | 0.978 |
| Adjusted $R^2$ | 0.658 | 0.838 | 0.973 |

*Note: 95% Confidence Interval in parentheses* — *p<0.1; **p<0.05; ***p<0.01

# Supporting Information 6: Estimates and confidence intervals of urban scaling coefficients in the ABM sample

We estimate the logarithm of adopters in towns with the logarithm of town population using an ordinary least squares regression. Table S3 details Figure 4C by reporting 95% confidence intervals for each estimates. All coefficients are significantly above 1. This indicates super-linear scaling meaning that adoption concentrates in large towns.

Table S3: Urban Scaling Coefficients

| | Innovators | Early Adopters | Majority and Laggards |
|---|---|---|---|
| Data | 1.34 | 1.26 | 1.06 |
| | (1.18,1.57) | (1.16,1.36) | (1.02,1.09) |
| DE | 1.19 | 1.18 | 1.04 |
| | (1.13,1.25) | (1.14,1.23) | (1.01,1.08) |
| ABM(h=0.0,l=0.0) | 1.12 | 1.10 | 1.10 |
| | (1.02,1.21) | (1.04,1.15) | (1.06,1.13) |
| ABM(h=0.2,l=0.2) | 1.22 | 1.13 | 1.08 |
| | (1.11,1.34) | (1.05,1.21) | (1.05,1.12) |

# Supporting Information 7: Assortativity of adoption fuels peak prediction bias in large towns

Connections of individuals with similar tendency to adopt, or assortative mixing, is crucial in spatial spreading. However, predicting the likelihood of adoption is the aim of diffusion models and a priori labeling of individuals in these models would be a paradox.

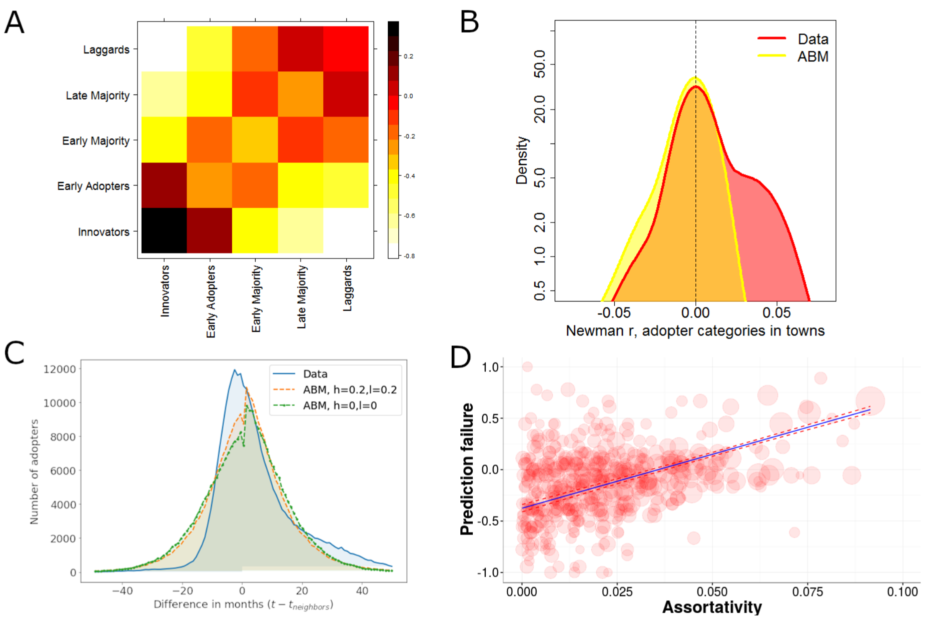


Figure S6: Assortativity in adoption and its bias in peak prediction. **A.** Assortative mixing of adoption categories. **B.** Assortative mixing (measured by Newman's r) is higher in the empirical data than in the ABM(h=0.0, l=0.0). **C.** Difference of adoption time (measured in months) between the user and his/her direct connections is smaller in the empirical data than in both versions of the ABM. **D.** Assortative mixing of adopter categories by Rogers correlates with the standardized Prediction Error in the town. Size of the dots denotes the log of town population; blue solid line represents predicted values from a linear regression with 95% confidence interval.

To illustrate adoption assortativity in our data in Figure S6A, we calculated the number of links between groups $W_{ij}$ and compared it to the expected number of ties $E(W_{ij})$ for which uniform distribution of links across the groups is assumed and is calculated by $\frac{\sum_j W_i * \sum_i W_j}{\sum W_i j}$. We have transformed the $\frac{W_i j}{E(W_i j)}$ ratio into the (-1; 1) interval using the $\frac{x-1}{x+1}$ formula. This indicator is positive if the observed number of ties exceeds the expected number of ties and negative otherwise. The plot suggests that assortative mixing fragment the network into categories of Innovators and Early Adopters who are only loosely connected to Late Majority and Laggard users.

To characterize assortativity on the town level, we classified each user into the adopter categories stated by Rogers[4] and calculated Newman's assortativity $r$ [5] for every town. This indicator takes the value of 0 when there is no assortative mixing by adopter types and a positive value when links between identical adopter types are more frequent than links between different adopter types. Figure S6B demonstrates the similarity of peers in each town using the Newman $r$ index of assortative mixing [5]. In many towns, the empirical data has a stronger assortativity than the ABM(h=0.0, l=0.0). This phenomenon is due to adoption time lag differences depicted in Figure S6C. Here we contrast ABM(h=0.0, l=0.0) and ABM(h=0.2, l=0.2) with empirical data in terms of the average difference between adoption time between each ego and the time of adoption of his/her network neighbors. The ABM differs from the empirical data in determining how fast individuals follow their connections. These observations confirm that assortative mixing in terms of adoption tendency is an important feature of spatial diffusion of innovation.

To test how assortative mixing influences the spatial prediction of the diffusion ABM(h=0.0, l=0.0) in Figure S6D, we estimated the prediction error with Newman's $r$ with ordinary least square estimator and used the number of OSN users in the town as weights in the regression. The ABM predicted adoption earlier in the majority of small towns, where no assortative mixing was found. On the contrary, the ABM predicted adoption late in large towns, where Innovators and Early Adopters were only loosely connected to Early- and Late Majority and Laggards. In case, we do not include weights in the regression, the point estimate of assortativity is not significant. These findings confirm that assortativity in terms of the adoption probability influences diffusion [6, 7] and fuels peak prediction bias in large towns.

# Supporting Information 8: Confidence intervals of Prediction Error estimations

We estimate the Prediction Error of DE, ABM(h=0.0, l=0.0) and ABM(h=0.2,l=0.2) models with town-level social network variables and geographical characteristics using ordinary least squares regressions. Table S4 details Figure 5C by reporting 95% confidence intervals for each estimates.

Table S4: Prediction Error Estimates

| | DE | ABM(h=0.0,l=0.0) | ABM(h=0.0,l=0.0) |
|---|---|---|---|
| Distance from Capital | 0.035 | -0.079 | -0.100 |
| | (0.021,0.048) | (-0.109,-0.048) | (-0.134,-0.065) |
| N. of Users | -0.011 | 0.020 | 0.023 |
| | (-0.015,-0.006) | (0.010,0.030) | (0.012,0.034) |
| Avg. Path Length | -0.017 | 0.026 | 0.026 |
| | (-0.024,-0.011) | (0.013,0.038) | (0.012,0.040) |
| Modularity | -0.065 | 0.074 | 0.098 |
| | (-0.089,-0.040) | (0.020,0.127) | (0.037,0.158) |
| Transitivity | -0.012 | -0.041 | 0.009 |
| | (-0.029,0.005) | (-0.076,-0.006) | (-0.029,0.049) |
| Density | 0.021 | -0.051 | -0.007 |
| | (0.007,0.035) | (-0.079,-0.023) | (-0.038,0.024) |

## Supporting Information 9: Regression table for ABM Prediction Error

To understand, whether Prediction Error of adoption peaks is governed by demographic characteristics of towns, we run multiple OLS regressions with Prediction Error of DE and ABM predictions as dependent variable. Independent variables include geographical variables that we focus on (population and distance) and further measures that have been used in previous studies to predict adoption rate, or to investigate inequalities: development level (average income[2]), inequalities (Gini of income[3]), internet infrastructure and media presence (Telecom Composite Index, Number of TV, Number of School PC[2]), physical barriers of social interaction in towns (Rail-River Division [3]), segregation (Ethnic Entropy[3]), town hierarchy (Subregion Centre[2]).

We find that Population and Distance influence ABM Prediction Error as reported in the main text. Also, prediction is slightly late in towns that are relatively developed (measured by average income). The rest of the socio-economic variables, however, do not have significant point estimates.

Table S5: Regression Results

| | *Dependent variable:* | | |
|---|---|---|---|
| | Pred. Fail., DE | Pred. Fail., ABM(h=0.0, l=0.0) | Pred. Fail., ABM(h=0.2, l=0.2) |
| | (1) | (2) | (3) |
| Population (log) | 0.039*** | 0.030*** | 0.038*** |
| | (0.023, 0.054) | (0.013, 0.047) | (0.019, 0.057) |
| Distance from Budapest, km (log) | −0.021 | −0.064*** | −0.076*** |
| | (−0.053, 0.011) | (−0.098, −0.029) | (−0.114, −0.038) |
| Average salary | −0.00002 | 0.0001 | 0.0001*** |
| | (−0.0001, 0.00004) | (−0.00001, 0.0001) | (0.00005, 0.0002) |
| Gini | 0.056 | 0.042 | 0.067 |
| | (−0.046, 0.159) | (−0.069, 0.153) | (−0.056, 0.190) |
| Telcom index | −0.001 | 0.004 | −0.003 |
| | (−0.013, 0.011) | (−0.009, 0.016) | (−0.017, 0.011) |
| TV use | −0.0002 | 0.0004 | 0.0001 |
| | (−0.001, 0.001) | (−0.0004, 0.001) | (−0.001, 0.001) |
| PC in school | 0.0002 | 0.00002 | 0.0003 |
| | (−0.0005, 0.001) | (−0.001, 0.001) | (−0.001, 0.001) |
| RRDI | 0.006 | −0.013 | 0.011 |
| | (−0.023, 0.036) | (−0.044, 0.019) | (−0.024, 0.046) |
| Ethnic entropy | −0.084 | −0.112* | −0.013 |
| | (−0.197, 0.028) | (−0.233, 0.010) | (−0.147, 0.122) |
| Town | 0.013 | 0.008 | −0.002 |
| | (−0.012, 0.039) | (−0.019, 0.036) | (−0.033, 0.028) |
| Constant | 0.075 | −0.029 | −0.117 |
| | (−0.295, 0.445) | (−0.431, 0.372) | (−0.561, 0.327) |
| Observations | 2,237 | 2,237 | 2,237 |
| $R^2$ | 0.026 | 0.030 | 0.038 |
| Adjusted $R^2$ | 0.014 | 0.017 | 0.025 |
| *Note:95% CI in parentheses.* | | | *p<0.1; **p<0.05; ***p<0.01 |

# References


[1] Xiao Y, Han JT, Li Z, Wang Z. A Fast Method for Agent-Based Model Fitting of Aggregate-Level Diffusion Data. SSRN; 2017. Available from: https://ssrn.com/abstract=2844202orhttp://dx.doi.org/10.2139/ssrn.2844202.

[2] Lengyel B, Jakobi Á. Online social networks, location, and the dual effect of distance from the centre. Tijdschrift voor economische en sociale geografie. 2016;107(3):298–315.

[3] Tóth G, Wachs J, Di Clemente R, Jakobi Á, Ságvári B, Kertész J, et al. Inequality is rising where social network segregation interacts with urban topology. arXiv preprint arXiv:190911414. 2019;.

[4] Rogers EM. Diffusion of innovations. Simon and Schuster; 2010.

[5] Newman ME. Mixing patterns in networks. Physical Review E. 2003;67(2):026126.

[6] Watts DJ. A simple model of global cascades on random networks. Proceedings of the National Academy of Sciences. 2002;99(9):5766–5771.

[7] Toole JL, Cha M, González MC. Modeling the adoption of innovations in the presence of geographic and media influences. PloS one. 2012;7(1):e29528.
